# Modulation of K_Ca_3.1 Channels by Eicosanoids, Omega-3 Fatty Acids, and Molecular Determinants

**DOI:** 10.1371/journal.pone.0112081

**Published:** 2014-11-05

**Authors:** Michael Kacik, Aida Oliván-Viguera, Ralf Köhler

**Affiliations:** 1 Faculty of Medicine, Philipps-University Marburg & Medical Center I, Clemenshospital/University Hospital of University Münster, 48153 Münster, Germany; 2 Aragon Institute of Health Sciences I+CS/IIS, 50009 Zaragoza, Spain; 3 Fundación Agencia Aragonesa para la Investigación y Desarrollo (ARAID), 50018 Zaragoza, Spain; Indiana University School of Medicine, United States of America

## Abstract

**Background:**

Cytochrome P450- and ω-hydrolase products (epoxyeicosatrienoic acids (EETs), hydroxyeicosatetraeonic acid (20-HETE)), natural omega-3 fatty acids (ω3), and pentacyclic triterpenes have been proposed to contribute to a wide range of vaso-protective and anti-fibrotic/anti-cancer signaling pathways including the modula-tion of membrane ion channels. Here we studied the modulation of intermediate-conductance Ca^2+^/calmodulin-regulated K^+^ channels (K_Ca_3.1) by EETs, 20-HETE, ω3, and pentacyclic triterpenes and the structural requirements of these fatty acids to exert channel blockade.

**Methodology/Principal Findings:**

We studied modulation of cloned human hK_Ca_3.1 and the mutant hK_Ca_3.1^V275A^ in HEK-293 cells, of rK_Ca_3.1 in aortic endothelial cells, and of mK_Ca_3.1 in 3T3-fibroblasts by inside-out and whole-cell patch-clamp experiments, respectively. In inside-out patches, Ca^2+^-activated hK_Ca_3.1 were inhibited by the ω3, DHA and α-LA, and the ω6, AA, in the lower µmolar range and with similar potencies. 5,6-EET, 8,9-EET, 5,6-DiHETE, and saturated arachidic acid, had no appreciable effects. In contrast, 14,15-EET, its stable derivative, 14,15-EEZE, and 20-HETE produced channel inhibition. 11,12-EET displayed less inhibitory activity. The K_Ca_3.1^V275A^ mutant channel was insensitive to any of the blocking EETs. Non-blocking 5,6-EET antagonized the inhibition caused by AA and augmented cloned hK_Ca_3.1 and rK_Ca_3.1 whole-cell currents. Pentacyclic triterpenes did not modulate K_Ca_3.1 currents.

**Conclusions/Significance:**

Inhibition of K_Ca_3.1 by EETs (14,15-EET), 20-HETE, and ω3 critically depended on the presence of electron double bonds and hydrophobicity within the 10 carbons preceding the carboxyl-head of the molecules. From the physiological perspective, metabolism of AA to non-blocking 5,6,- and 8,9-EET may cause AA-de-blockade and contribute to cellular signal transduction processes influenced by these fatty acids.

## Introduction

The intermediate-conductance Ca^2+^/calmodulin-activated K^+^ channel, K_Ca_3.1 (encoded by the *KCNN4* gene) produces K^+^-efflux and cell membrane hyperpolarization to mobilization of intracellular Ca^2+^ [1], [2], [3]. The channel is mainly expressed in red and white blood cells [4], [5], [6], secretory epithelia of salivary glands [7], intestine [8], bronchioles [9], vascular endothelium [10], proliferating smooth muscle [11], [12], [13], [14] and fibroblasts [15], [16], and malignant brain cancers ([17], [18], for review see [19], [20]. In these tissues, the channel contributes to the regulation of cell volume [4], anion and water secretion [8], cytokine production [21], endothelial vasodilator responses [10], Ca^2+^-dependent cell cycle progression, cell migration, and mitogenesis [14], [22], [23], respectively.

At the molecular level, the most important determinant of channel activation is an increase of intracellular Ca^2+^ that causes conformational changes of constitutively bound calmodulin [1], [2], leading to channel gating. Besides this principal mechanism, c-terminal phosphorylation of the channel by cAMP/PKA-dependent mechanisms [24] has been proposed to cause endogenous positive-regulation of channel activity. The omega-6 fatty acid (ω6), arachidonic acid (AA), was identified by Dan Devor and coworkers as the first negative endogenous regulator of K_Ca_3.1 [25]. Moreover, their seminal work revealed also major mechanisms of membrane trafficking and internalization/recycling/degradation of hK_Ca_3.1 [26], [27]. AA-inhibition of the channel is presumably caused by AA-interaction with lipophilic residues (T250/V275) lining the channel cavity below the selectivity filter and presumed gate of K_Ca_3.1 [25]. Yet, the structural requirements of the fatty acid itself for K_Ca_3.1-blockade are unknown.

Here, we hypothesized that structurally related omega-3 fatty acids (ω3), docosahexaenoic acid (DHA) and α-linolenic acid (α-LA), the cytochrome-P450-epoxygenase (CYP)-generated metabolites of AA, epoxyeicotrienoic acids (5,6-EET, 8,9-EET, 11,12-EET, and 14,15-EET) as well as the ω-hydroxylase product, 20-hydroxyeicosatetraeonic acid (20-HETE), are additional lipid modulators of K_Ca_3.1. Moreover, epoxygenation of AA to 5,6-EET, 8,9-EET, 11,12-EET, or 14,15-EET may shed light on the structural requirements for channel modulation. In addition, a potential K_Ca_3.1-regulation by EETs, 20-HETE, and ω3 could be of help to understand the physiological actions of these fatty acids in physiological systems like the vascular endothelium and arteries, in which they have been shown to exert vasodilator or vasoconstrictor actions, respectively (for review see [28], [29], [30]). Moreover, EETs and ω3 have been proposed to have anti-inflammatory and anti-atherosclerotic activity and to modulate angiogenesis, cardiac fibrosis and cancer growth [31], [32], [33], [34], [35]. In this respect, EETs and K_Ca_3.1-functions have overlapping impacts and may be mechanistically linked as components of the same signal transduction pathway(s). Today, several downstream targets such as G-protein-coupled receptors have been proposed to mediate EET-actions but specific receptors for EETs, HETEs, as well as for ω3 are still elusive (for review see [30], [31]). So far it is unknown whether these fatty acids modulate hK_Ca_3.1-functions.

In addition to these fatty acids, we tested whether lipids of the pentacyclic triterpene class, uvaol, erythrodiol, oleanolic acid, and maslinic acid, exert K_Ca_3.1-modulatory actions. These natural triterpenes are found in virgin olive oil and have been suggested having antioxidant, antifibrotic, anti-atherosclerotic, and, both, pro- as well as anti-inflammatory activities [35], [36], [37], [38]. However, whether these actions are related to - at least in part - K_Ca_3.1-modulation has not been studied before.

**Figure 1:**
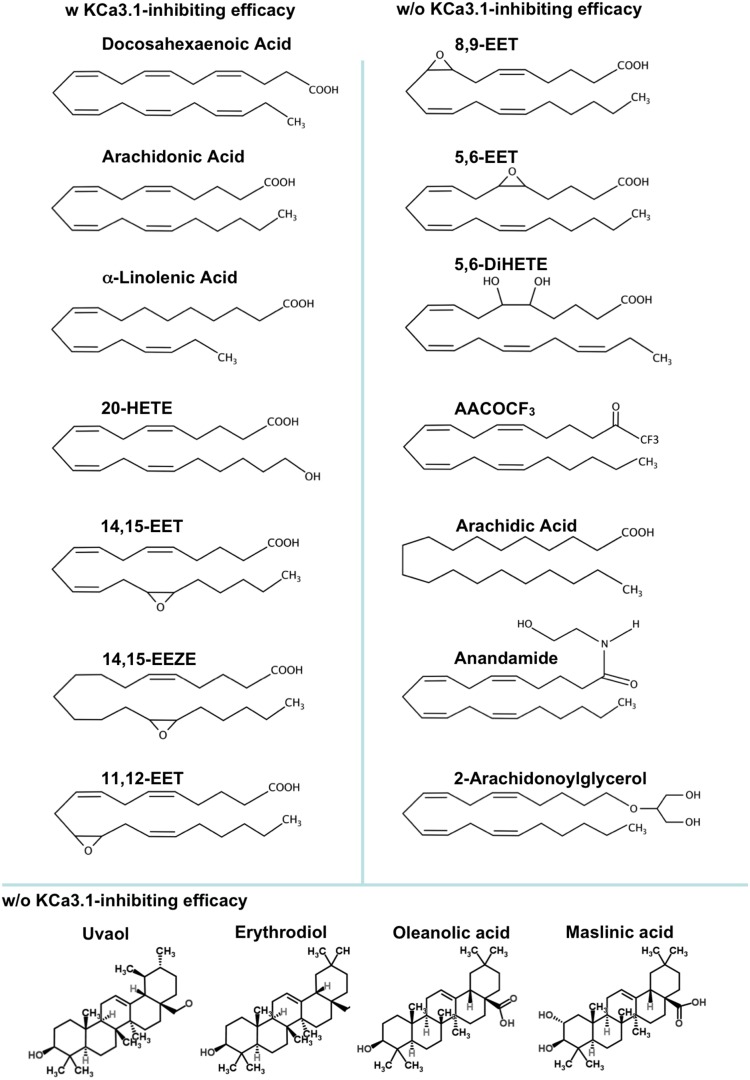
Chemical structures of eicosanoids, ω3, and pentacyclic triterpenes and schematic overview of blocking efficacy (decreasing from top to bottom) or non-blocking efficacy.

We therefore conducted an electrophysiological study on cloned hK_Ca_3.1 and endothelial rK_Ca_3.1 and studied channel modulation by selected ω3, the four EETs, and 20-HETE, synthetic stable analogues, and other related fatty acids with structural differences or similarities (for structures see Figure 1). To further study potential binding/interaction sites within the K_Ca_3.1 channel, we investigated blocking efficacy of the fatty acids on the AA-insensitive K_Ca_3.1-mutant V275A [25]. Moreover, we studied the interactivity of EETs with its precursor, AA. In murine fibroblast, we tested the modulation of mK_Ca_3.1 by DHA and by pentacyclic triterpenes.

Our major findings were that the 14,15-EET, 20-HETE, DHA, and α α-LA, were negative modulators of K_Ca_3.1 while non-blocking 5,6-EET antagonized AA-mediated inactivation. KCa3.1 blockade critically depended on hydrophobicity of the 10 carbons preceding the carboxyl head and the presence of at least one electron double bond in this part of the carbon chain.

## Materials and Methods

### Cells, channel clones, and cell culture

HEK-293 cells stably expressing hK_Ca_3.1 were a kind gift from Dr. Khaled Houamed, University of Chicago and Dr. Heike Wulff, Department of Pharmacology, University of California, Davis. Stably expressing cells were selected with puromycin (1 µg/ml; Sigma, Deisenhofen, Germany). The hK_Ca_3.1^V275A^, hK_Ca_3.1^T250S^, and hK_Ca_3.1^T250S/V275A^ mutants were kind gifts from Dr. Dan Devor, University of Pittsburgh, Department of Cell Biology. The clones were stably expressed in HEK-293 using FuGENE 6 Transfection kit (Roche, Basel, Switzerland) and manufacturer’s protocols. Stably expressing HEK-293 cells were selected using geneticin (G-418, 100 µl/10 ml; Sigma, Deisenhofen, Germany). Rat aortic endothelial cells with endogenous rK_Ca_3.1 were provided by the BMFZ of the Philipps-University Marburg [39]. Murine 3T3 fibroblasts were obtained from ATCC (3T3-L1, ref# CL-173, ATCC, Rockville, MD). As usual cell culture medium, we used Dulbecco’s Modified Eagle Medium (DMEM) supplemented with 10% calf serum and 1% penicillin/streptomycin (all from Biochrom KG, Berlin, Germany). Before patch-clamp, cells were trypsinized and seeded on cover slips for 4–24 hrs.

### Patch-clamp electrophysiology

Membrane currents in excised inside-out patches and whole-cell currents were recorded with an EPC-9 patch-clamp amplifier (HEKA, Lambrecht Pfalz, Germany) using borosilicate glass pipettes with a tip resistance of 2–3 MOhm. Seal resistance was above 1 GOhm. In inside-out experiments, we continuously monitored outward currents at a holding potential of 0 mV prior to patch excision and thereafter. Activation of K_Ca_3.1-mediated currents occurred immediately after excision of the patch and exposure of the intracellular side of the patch to the Ca^2+^-containing bath solution (“intracellular” solution see below). For conventional whole-cell current measurements, we used voltage ramps (voltage range for recording: −120 mV to +100 mV; duration, 1 sec; applied every 3 sec; voltage range evaluated: −110 to +30 mV). Series resistance was between 7–15 MegaOhms and membrane resistance was >1 GigaOhm. In such experiments, the “intracellular” Ca^2+^-containing solution was “infused” into the cell via the patch-pipette after seal rupture activating K_Ca_-currents usually within 2–10 sec. Current amplitudes remained stable thereafter over 5 min and longer in some. The solution was composed of (mM): 140 KCl, 1 MgCl_2_, 1 Na_2_ATP, 2 EGTA, 1.92 CaCl_2_ (3 µM [Ca^2+^]_free_) and 5 HEPES (adjusted to pH 7.2 with KOH). In a subset of experiments, [Ca^2+^]_free_ was buffered to 0.01, 0.3, 0.5 µM [Ca^2+^]_free_ (0,07, 0.72, 1.25, and 1.48 mM CaCl_2_, each combined with 2 mM EGTA). The “extracellular” solution was composed of (mM): 137 NaCl, 4.5 Na_2_HPO_4,_ 5 KCl, 1.5 KH_2_PO_4_, 1 MgCl_2_, 1 CaCl_2_, 10 EGTA (10 nM [Ca^2+^]_free_), 10 glucose and 10 HEPES (adjusted to pH 7.4 with NaOH). For additional details, see [16]. In inside-out experiments, the high Na^+^ solution served as pipette solution and the high K^+^ solution as bath solution; in whole-cell experiments, vice versa. For measurements of rK_Ca_3.1 currents in RAEC, we performed the experiments in the presence of the K_Ca_2 blocker UCL-1684 (250 nM) [40] to eliminate rK_Ca_2.3 currents in these cells.

### Chemicals and drugs

Standard chemicals were obtained from Sigma-Aldrich (Deisenhofen, Germany). 5,6-EET (4-{3-[(2Z,5Z,8Z)-tetradeca-2,5,8-trien-1-yl]oxiran-2-yl}butanoic acid), 8,9-EET ((5Z)-7-{3-[(2Z,5Z)-undeca-2,5-dien-1-yl]oxiran-2-yl}hept-5-enoic acid), 11,12-EET ((5E,8Z)-10-{3-[(2E)-oct-2-en-1-yl]oxiran-2-yl}deca-5,8-dienoic acid), 14,15-EET ((5Z,8Z,11Z)-13-(3-pentyloxiran-2-yl)trideca-5,8,11-trienoic acid), 5,6-DiHETE ((8Z,11Z,14Z)-5,6-dihydroxy-8,11,14-icosatrienoic acid), 14,15-EEZE ((5Z)-13-[(2S,3R)-3-pentyl-2-oxiranyl]-5-tridecenoic acid), and 20-HETE ((5Z,8Z,11Z,14Z)-20-hydroxy-5,8,11,14-icosatetraenoic acid) were purchased from Cayman Chemicals (Michigan, IL, USA). Arachidonic acid ((5Z,8Z,11Z,14Z)-5,8,11,14-icosatetraenoic acid), arachidonyl glycerol (1,3-dihydroxy-2-propanyl (5Z,8Z,11Z,14Z)-5,8,11,14-icosatetraenoate), arachidic acid (icosanoic acid), charybdotoxin, docosahexaenoic acid ((4Z,7Z,10Z,13Z,16Z,19Z)-4,7,10,13,16,19-docosahexaenoic acid), α-linolenic acid ((9Z,12Z,15Z)-9,12,15-octadecatrienoic acid), dimethyl sulfoxide (DMSO) and acetonitrile were obtained from Sigma-Aldrich. Arachidonyl trifluoromethyl ketone (AACOCF_3_; (6Z,9Z,12Z,15Z)-1,1,1-trifluoro-6,9,12,15-henicosatetraen-2-one), anandamide ((5Z,8Z,11Z,14Z)-N-(2-hydroxyethyl)-5,8,11,14-icosatetraenamide), and UCL-1684 (17,24-diaza-1,9-diazoniaheptacyclo[23.6.2.29,16.219,22.13,7.010,15.026,-31]octatriaconta-1(31),3(38),4,6,9,11,13,15,19,21,25,27,29,32,34,36-hexadecaene) were obtained from TOCRIS (Germany). Uvaol ((3β)-Urs-12-ene-3,28-diol), erythrodiol ((3β)-Olean-12-ene-3,28-diol), oleanolic acid ((3β)-3-Hydroxyolean-12-en-28-oic acid), and maslinic acid ((2α,3β)-2,3-Dihydroxyolean-12-en-28-oic acid) were kind gifts from Dr. Jesús Osada, Department of Biochemistry and Molecular and Cellular Biology, Veterinary School, Health Research Institute of Aragon, CIBEROBN, Zaragoza, Spain. EETs were delivered as ethanol stock solutions. Ethanol was evaporated under nitrogen stream and the EETs were reconstituted in DMSO at a concentration of 10 mM. Stocks were stored at −20°C until use. Stock solutions of the other fatty acids (10 mM) were also prepared with DMSO. Ahead of use stock solutions were diluted 1∶10 with the bath buffer and the final DMSO concentration did not exceed 0.2%. Since unsaturated fatty acids are sensitive to oxidative degradation, we minimized exposure times in aqueous solutions and to air and prepared the aqueous pre-dilutions of the compounds immediately before starting the experiments. Bath solutions were not gassed with oxygen.

### Statistics

Data are given as mean ± SEM. For statistical comparison of multiple data sets we used one-way ANOVA and the Tukey *post hoc* and p-values of <0.05 were considered significant.

## Results

**Figure 2:**
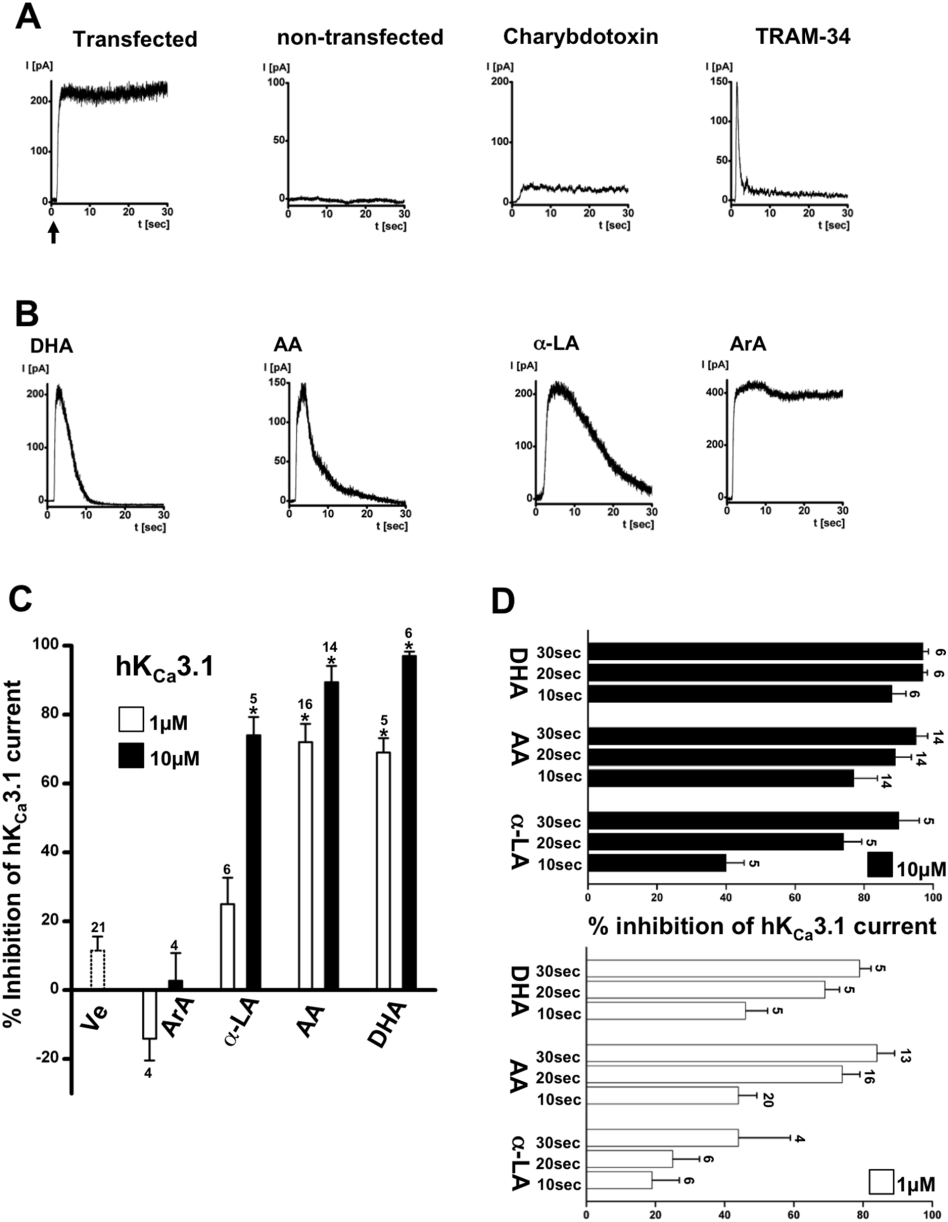
Membrane expression of cloned human K_Ca_3.1 in HEK-293 in inside-out patches and basic pharmacological characterization. A) From left to right: Exemplary traces of immediate activation of hK_Ca_3.1-outward currents upon excision of the patch into 3 µM Ca^2+^-containing bath solution (as indicated by arrow). K_Ca_-outward currents are absent in non-transfected HEK-293. Inhibition of hK_Ca_3.1-outward currents by charybdotoxin (100 nM, in the pipette solution) and TRAM-34 (1 µM, in the bath solution). B) Inhibition of hK_Ca_3.1 by ω3 and arachidonic acid. From left to right: Time course of inactivation of hK_Ca_3.1 by docosahexaenoic acid (DHA, 10 µM), arachidonic acid (AA, 10 µM), α-linolenic acid (α-LA, µM) over time. Saturated arachidic acid (ArA, 10 µM) did not affect channel activity. C) Concentration-dependence of inhibition. Note that half of the current was inhibited by AA, DHA, and α-LA at approx. 1 µM. D) Time course of channel inactivation by two concentrations of AA, DHA, and α-LA over time. Data are means ± SEM (% inhibition of K_Ca_3.1-current normalized to initial peak amplitude after patch-excision); numbers in the graphs indicate the number of inside-out experiments; *P<0.05 vs. vehicle (Ve); One-way ANOVA and Tukey *post hoc* test.

In inside-out experiments on HEK-293 expressing cloned hK_Ca_3.1, excision of the patch into the 3 µM Ca^2+^-containing bath solution caused immediate activation of K^+^-outward currents that were stable over several minutes (Figure 2A). Non-transfected cells did not display these currents. In hK_Ca_3.1-HEK-293, K^+^-outward currents were virtually absent in the continuing presence of the classical K_Ca_3.1-blocking toxin, charybdotoxin, in the “extracellular” pipette solution (Figure 2A). Likewise, in the continuing presence of the selective small molecule blocker of K_Ca_3.1, TRAM-34 [6], in the bath solution prevented K^+^-outward currents, although we observed an initial spike-like outward current (Figure 2A) after excision of the patch.

In the continuing presence of 1 or 10 µM of the ω3, docosahexaenoic acid (DHA), arachidonic acid (AA), and α-linolenic acid (α-LA), hK_Ca_3.1 currents could still be activated by patch-excision but the currents did not last and were inhibited after 30 sec (Figure 2B and C). The inhibition by 1 µM was less pronounced than inhibition by 10 µM for all ω3 tested here (Figure 2C). However, potencies and kinetics of current inhibition differed between the ω3 with the following order of potency and time to full inhibition: DHA≥AA>α-LA (Figure 2D). In contrast, the saturated fatty acid, arachidic acid (ArA), did not produce channel inhibition (Figure 2B and C).

**Figure 3:**
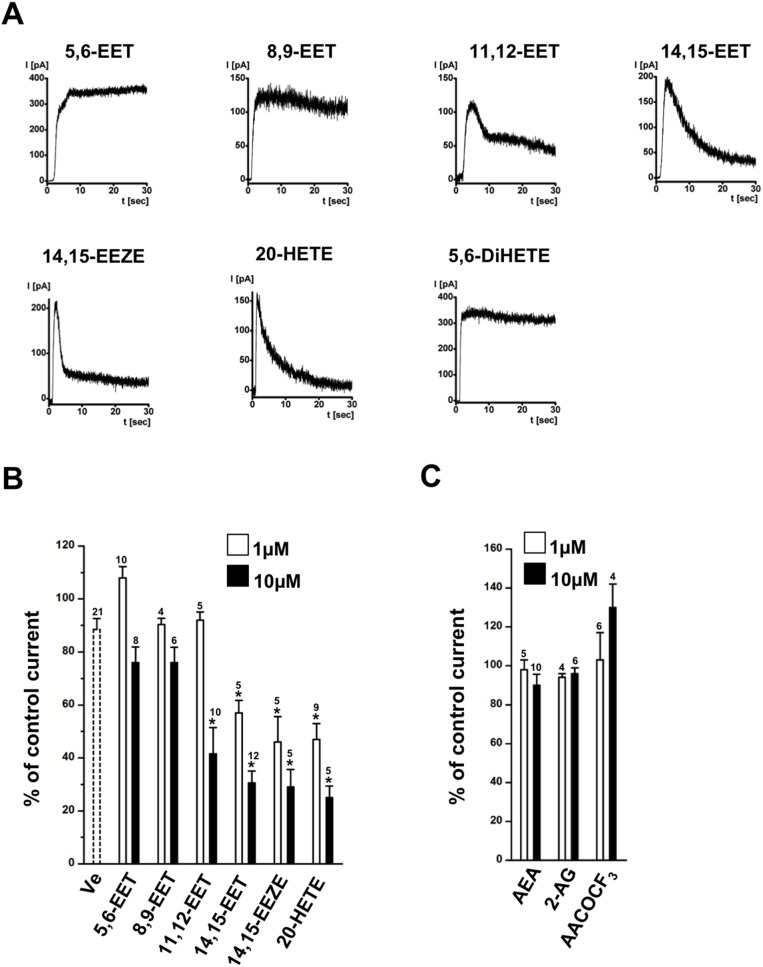
Heterogeneous sensitivity of hK_Ca_3.1 to the four EETs, stable 14,15-EEZE, 20-HETE and 5,6-DiHETE. A) Representative traces of hK_Ca_3.1 outward-currents in inside-out patches overtime in the continuing presence of the fatty acids at 10 µM. B) Summary data of maximal change of current (% of control) at two concentrations (1 and 10 µM). 5,6 DiHETE was tested at 10 µM (0±10%, n = 4). C) No K_Ca_3.1-blockade in the presence of anandamide (AEA; 10 µM), arachidonoylglycerol (2-AG; 10 µM), arachidonyl trifluoromethyl ketone (AACOCF_3_; 10 µM). Numbers in the graphs indicate the number of inside-out experiments. Data are means ± SEM (% inhibition of K_Ca_3.1-current normalized to initial peak amplitude after patch-excision); *P<0.05 vs. vehicle (Ve); One-way ANOVA and Tukey *post hoc* test.

With respect to the four EETs, 5,6-EET, 8,9-EET, 11,12-EET, 14,15-EET (Figure 3A for current traces and B for summary data), we found that only 14,15-EET displayed substantial inhibition with potency and kinetics similar to those observed with α-LA. 11,12-EET produced less inhibition. 5,6-EET, 8,9-EET, and 5,6-DiHETE produced virtually no inhibition. The stable analogue of 14,15-EET, 14,15-EEZE, produced an inhibition similar to that caused by 14,15-EET. The ω-hydroxylase product, 20-HETE that was hydroxylated at C20 (end) of the carbon chain, inhibited the current with kinetics and potency similar to other blocking fatty acids (Figure 3A and B). In contrast, molecules that differed from EETs and ω3 because of a major modification of the carboxyl group to hydroxyethylamide like in arachidonoyl ethanolamide (AEA), also known as anandamide, to a 1,3-dihydroxy-2-propanyl as in 2-arachidonoylglycerol (2-AG), and to trifluoromethyl ketone as in arachidonyl trifluoromethyl ketone (AACOCF_3_) did not produce inhibition (Figure 3C).

**Figure 4:**
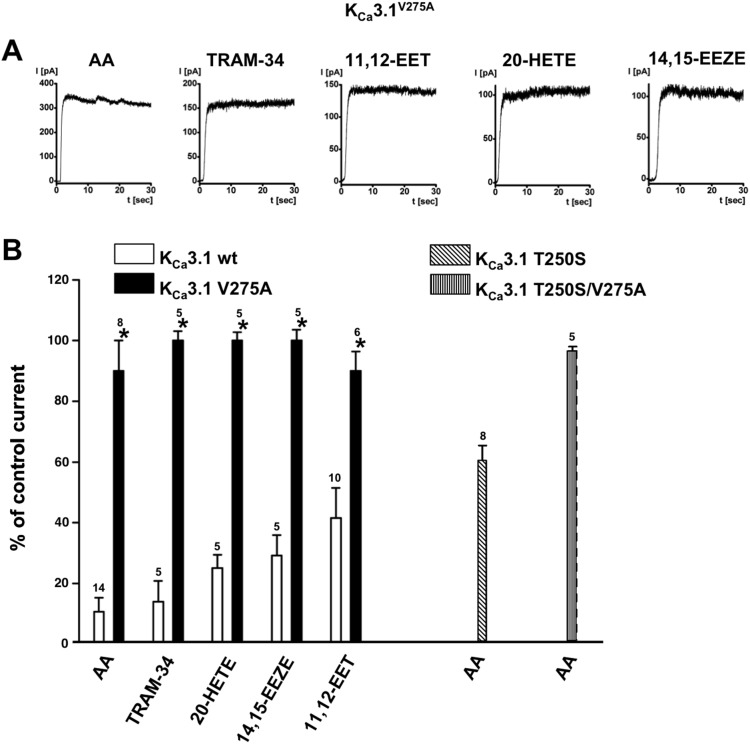
Insensitivity of hK_Ca_3.1 mutants. A) Representative current traces obtained from inside-out recordings using HEK-293 expressing the hK_Ca_3.1^V275A^ mutant. B) Summary data from experiments using the three different hK_Ca_3.1 mutants and wt hK_Ca_3.1. Concentration of all compounds was 10 µM. Data are means ± SEM; numbers in the graphs indicate the number of inside-out experiments. *P<0.05 vs. wt; One-way ANOVA and Tukey *post hoc* test.

The single mutants, hK_Ca_3.1^V275A^ and hK_Ca_3.1^T250S^, and the double mutant, hK_Ca_3.1^V275A/T250S^, were largely insensitive to AA and TRAM-34 (data shown for hK_Ca_3.1^V275A^), although the hK_Ca_3.1^T250S^ mutant appeared to have a smaller impact compared to the virtually complete insensitivity of the hK_Ca_3.1^V275A^ mutant to AA (Figure 4A and B). With respect to the other hK_Ca_3.1-blocking fatty acids, hK_Ca_3.1^V275A^ mutant was also insensitive to 11,12 EET, 14,15-EEZE, and 20-HETE as examples of fully (14,15-EEZE, 20-HETE) or partially (11,12-EET) hK_Ca_3.1-blocking fatty acids (Figure 4A and B).

**Figure 5:**
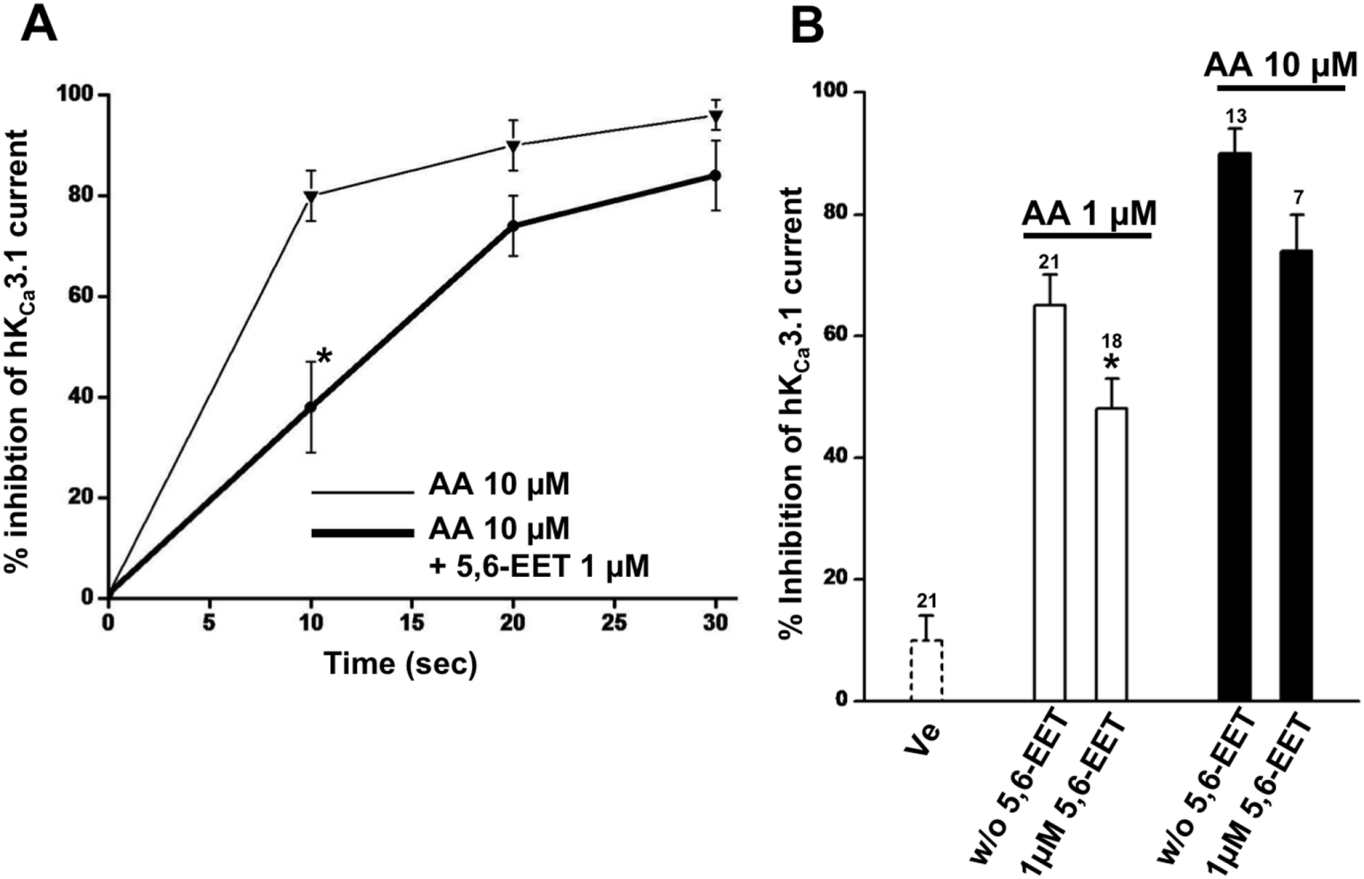
Moderate antagonism of AA-mediated hK_Ca_3.1-inhibtion by 5,6-EET. A) Time course of channel inhibition by 10 µM of AA in the presence of 1 µM 5,6-EET. B) Summary data of channel inhibition at 20 s after seal excision and with two concentrations (1 and 10 µM) of 5,6-EET and AA. Data are means ± SEM; numbers in the graphs indicate the number of inside-out experiments; *P<0.05 vs. AA alone, One-way ANOVA and Tukey *post hoc* test.

We next tested the idea whether the 5,6-EET as a non-blocking EET antagonizes AA-mediated channel blockade. These experiments showed that in the presence of both fatty acids, 1 µM 5,6-EET did not significantly prevent channel inhibition by 10 µM AA although the time period to achieve channel inhibition appeared to be increased (Figure 5A and B). At 1 µM AA we observed a significant antagonism of channel blockade by 1 µM 5,6-EET at a later time point (Figure 5A and B).

**Figure 6:**
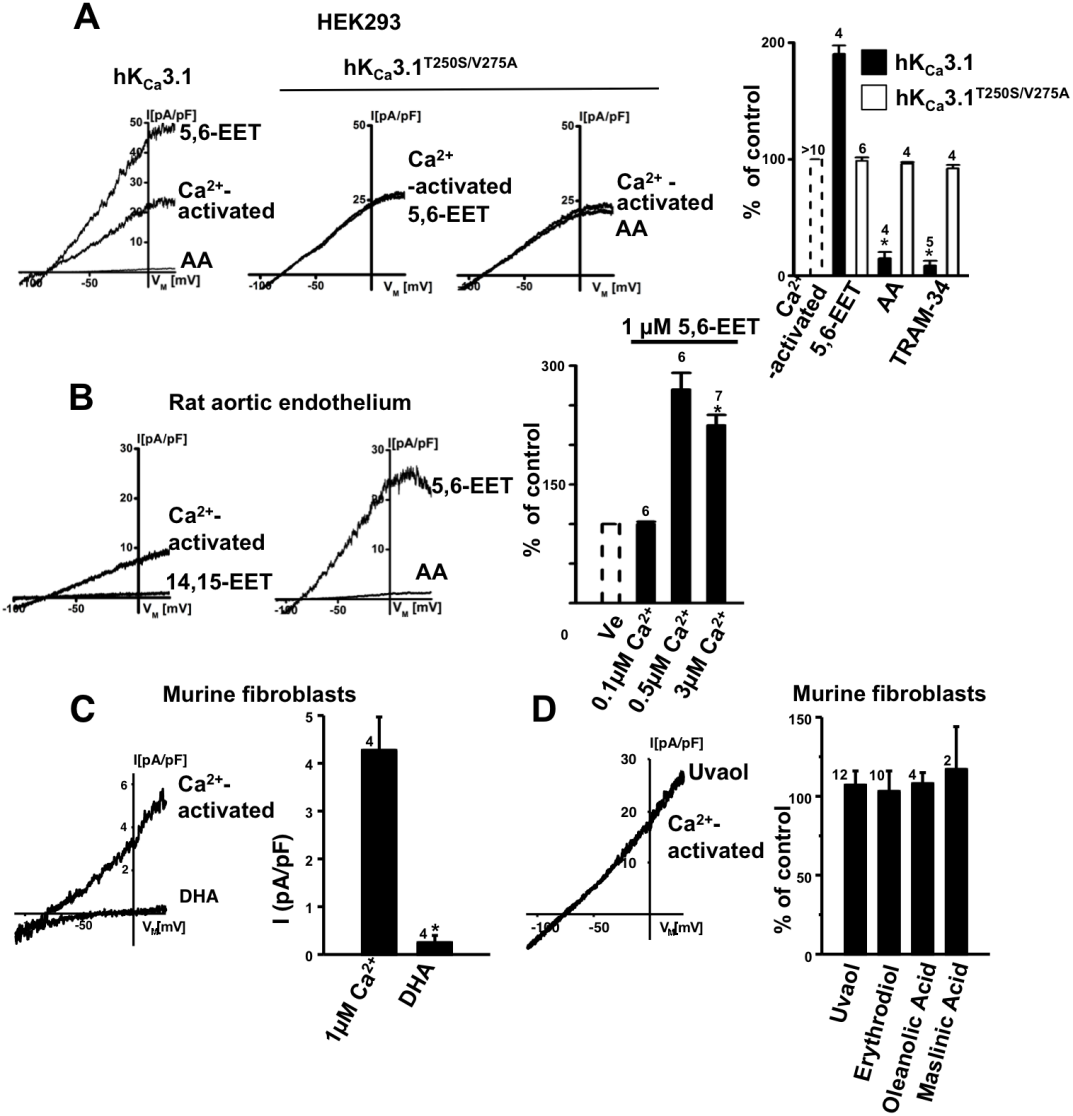
5,6-EET-potentiation of K_Ca_3.1 currents. A) Whole-cell current traces; from left to right: potentiation of Ca^2+^-pre-activated hK_Ca_3.1 by 5,6-EET (1 µM) followed by inhibition of the current by AA (10 µM), insensitivity of the hK_Ca_3.1^T250S/V275A^ mutant to 5,6-EET, and insensitivity of the hK_Ca_3.1^T250S/V275A^ mutant to AA (10 µM) and TRAM-34 (1 µM). The hK_Ca_3.1 currents were pre-activated by 250 nM Ca^2+^. Panel on the right: summary data. B) From left to right: Ca^2+^-pre-activation of rat endothelial rK_Ca_3.1 by 3 µM Ca^2+^ and current inhibition by 14,15-EET (1 µM), larger currents in the presence of 5,6-EET (1 µM) and inhibition by AA (10 µM). Panel on right: Summary data: dependence of 5,6-EET-potentiation on the intracellular Ca^2+^. Note that at a low intracellular Ca^2+^ (0.1 µM) that is below/near the threshold for K_Ca_3.1 activation, 5,6-EET did not potentiate the current. In contrast, potentiation occurred at an intracellular Ca^2+^ concentration that is near the EC_50_ for Ca^2+^-activation of K_Ca_3.1 as well as at a saturating Ca^2+^ concentration. C) DHA (1 µM) blocked Ca^2+^-pre-activated mK_Ca_3.1 in murine fibroblasts. D) Pentacyclic triterpenes did not modulate murine fibroblast mK_Ca_3.1 at a concentration of 1 µM. Data are means ± SEM (% inhibition of K_Ca_3.1-current normalized to initial peak amplitude after establishing electrical access (by seal rupture) and stable Ca^2+^-activation of K_Ca_3.1-outward currents); Numbers in the graphs indicate the number of whole-cell experiments; *P<0.05 vs. control (peak amplitude of the K_Ca_3.1-current in the respective cell); One-way ANOVA and Tukey *post hoc* test.

An increase of intracellular Ca^2+^ stimulates Ca^2+^-dependent PLA_2_ activity and AA-release. In our fast-whole cell experiments using a pipette solution with 0.3 µM Ca^2+^_free_, we expected Ca^2+^-dependent activation of hK_Ca_3.1 and also Ca^2+^-dependent PLA-2-mediated AA-release. In keeping with the idea that 5,6-EET antagonizes endogenous AA effects, we hypothesized that 5,6-EET augments total hK_Ca_3.1-currents in the HEK-293 cells and tested this in a small series of fast-whole cell experiments (Figure 6). We found that 5,6-EET (at 1 µM) produced significant potentiation by ≈twofold of the K_Ca_3.1 current that was pre-activated by 0.3 µM intracellular Ca^2+^ (Figure 6A). A high concentration of AA (10 µM) abolished these 5,6-EET-potentiated currents. Whole-cell currents produced by the hK_Ca_3.1^T250S/V275A^ mutant did not show potentiation by 5,6-EET (Figure 6A, right panel).

We performed another series of whole-cell experiments on rat aortic endothelial cells (RAEC) as an established and physiologically relevant cell system involving Ca^2+^-dependent AA and CYP/EETs signaling as well as K_Ca_3.1-dependent hyperpolarization as two mechanisms for endothelium-dependent vasodilation besides the nitric oxide pathway [29]. We tested specifically whether 1) AA and 14,15-EET produced a similar inhibition of endogenous rK_Ca_3.1 channels in RAEC, 2) rK_Ca_3.1 currents displayed a similar sensitivity to inhibition by AA, and 3) 5,6-EET produced potentiation of the current. As shown in figure 6B, these experiments revealed that 14,15-EET at 1 µM abolished calcium-activated rK_Ca_3.1 currents in these RAEC, in this regard similar to the findings in hK_Ca_3.1-overexpressing HEK-293. With respect to 5,6-EET-potentiation we found that 5,6-EET at 1 µM potentiated by ≈2.5-fold these endothelial calcium-activated rK_Ca_3.1 currents being pre-activated by 0.5 µM and 3 µM intracellular Ca^2+^ but not at 0.1 µM, a Ca^2+^-concentration that did not allow channel pre-activation (Figure 6B). AA at a concentration of 10 µM substantially blocked this 5,6-EET-potentiated current. Similar to the inside-out experiments, we did not see appreciable antagonistic effects at this lower concentration (1 µM) of 5,6-EET in these whole-cell experiments.

The ω3, DHA, and pentacyclic triterpenes as e.g. uvaol have been demonstrated experimentally to protect against cardiac fibrosis [35], [36], in addition to their documented vaso-protective and anti-inflammatory actions [37], [38]. Recently, we reported membrane expression of K_Ca_3.1 channels in proliferating murine 3T3-fibroblasts [16]. In the present study, we performed a series of whole-cell experiments and tested whether DHA and pentacyclic triterpenes inhibited mK_Ca_3.1 in murine fibroblasts. We found that DHA at 1–10 µM abolished virtually mK_Ca_3.1 (Figure 6C). In contrast, the pentacyclic triterpenes, uvaol, erythrodiol, maslinic acid, and oelanic acid, did not modulate mK_Ca_3.1-currents at 1 µM (Figure 6D).

## Discussion

Here we studied modulation of K_Ca_3.1 channel by CYP-products, 5,6-EET, 8,9-EET, 11,12-EET, and 14,15-EET, the ω-hydrolase product, 20-HETE, and the ω3, DHA, and α-LA, and identified structural requirements of these fatty acids for K_Ca_3.1-modulation. Our major findings were that 14,15-EET and 20-HETE as well as DHA and α-LA produced K_Ca_3.1 inhibition with potencies in the lower µmolar range. 11,12-EET was less potent and 5,6-EET and 8,9-EET did not cause inhibition. However, 5,6-EET was able to antagonize AA-induced inhibition. The observation that 14,15-EET and 20-HETE were efficient inhibitors while 5,6 and 8,9-EET not, identified the hydrophobic carbon stretch from C1–10 of the carboxyl head of the molecule as structural requirement for channel inhibition (for schematic overview of structural features of K_Ca_3.1-blocking and non-blocking fatty acids see Figure 1).

Several down-stream targets and receptors for propagation of intracellular or paracrine actions of EETs and ω3 have been proposed and, particularly, ion channel modulation by these fatty acid emerged as an additional mechanistic step. Yet, a plethora of channels have been shown to be directly activated by EET or to be a downstream target of EETs [41], [42], [43], [44], [45], [46], [47]. For instance, the TRPV4 channel, a member of the transient receptor potential gene family of cation channels, have been proposed to be activated by 5,6-EET and 8,9-EET and the resulting Ca^2+^-influx into the vascular endothelium caused vasorelaxation [43], [44]. TRPA1 channels in afferent neurons were activated by 5,6-EET leading to an increase in nociception in mice [48]. Yet, another TRP channel, the TRPC6 channel, has been shown to be translocated in a PKA-dependent manner to the cell membrane that required 11,12-EET binding to Gs-receptors in endothelial cells [49]. Moreover, 11,12-EET has been proposed to induce hypoxic vasoconstriction in the lung involving TRPC6 mechanism [50]. Other studies showed that 14,15-EET mediates phosphorylation of epithelial sodium channel (ENAC) activity in an ERK1/2 dependent mechanism [51].

With respect to K^+^ channels, 8,9-EET, 11,12-EET, and 14,15-EET have been reported to activate ATP-sensitive K^+^- channels by allosteric interaction with the ATP-binding site of the channel [52]. Two-pore tandem K^+^ channels (K2P) and large-conductance K_Ca_1.1 channels were known since long to be activated by ω3 and ω6 [45], [46], [53], [54], [55]. Moreover, 11,12-EET activation of K_Ca_1.1 channels was considered a main mechanism in smooth muscle, by which EET produced vasorelaxation [56]. In contrast, 20-HETE has been shown recently to enhance angiotensin-II-induced vasoconstriction by inactivating K_Ca_1.1 channels [57]. Interestingly, AA has also been shown to inhibit voltage-gated K^+^ channels such as the T lymphocyte KV1.3 channel [58] and the endogenous KV channels in HEK-293 (unpublished observation by our group). To our knowledge there were no data on direct or downstream modulation of K_Ca_3.1 channels by EETs that were not simply linked to EET-mediated increase in intracellular Ca^2+^. Hence, it was well established that K_Ca_3.1 channels were inhibited by the ω6, AA, that required mechanistically interaction with the lipophilic residues, V275 and T250, lining the channel cavity [25]. The structural requirements of the AA molecule to produce this inhibition remained however unclear. Our present study confirmed AA-mediated inhibition and the requirements of residue V275 and to some extent of T250 (Figure 4). Moreover, we provided additional insight by showing that the ω3, DHA and **α**-LA produced similar inhibition of the cloned human channel. Moreover, we showed here that AA abolished endogenous rK_Ca_3.1 (Figure 6) that suggested that AA could be an endogenous negative regulator of K_Ca_3.1 in the endothelium and could thereby influence the K_Ca_3.1-dependent endothelium-derived hyperpolarization (EDH)-mediated type of arterial vasodilation [28], [59]. However, this has not been further clarified by the present study. Interestingly, our inside-out experiments showed that K_Ca_3.1 could still be activated in the continuous presence of the AA but inactivated rapidly following Ca^2+^-dependent activation (Figure 2). This suggested a major impact of AA on K_Ca_3.1-gating unlike charybdotoxin (Figure 2) that obstructs simply the pore and ion flow by binding to the outer vestibule of the channel, independently of gating. However, we cannot exclude that this transient activation seen in the presence of AA reflected a delay of inhibition caused by diffusion of AA and the other compounds from the bath solution towards the excised membrane patch in the patch pipette.

With respect to eicosanoid-modulation of K_Ca_3.1, our study demonstrated that 14,15-EET, the stable analogue, 14,15-EEZE, and 20-HETE were K_Ca_3.1-inhibitors with potencies slightly below that of AA. Structurally, this inhibition required apparently hydrophobicity and 2 double electron bonds within the first 10 carbons of the carboxyl head of the molecules. This was concluded from the lack of inhibitory activity of 5,6-EET and 8,9-EET, in which this part of the fatty acid chain was epoxygenated. The partial inhibition caused by 11,12-EET could be explained by the conserved hydrophobicity within carbons 1–10 although 11,12-epoxygenation appeared to have efficacy-reducing impact. In respect to channel-eicosanoids interactions, it was likely that epoxygenation as in 5,6,-EET and 8,9-EET did not allow the proper interactions of these molecules with hydrophobic residues of the cavity below the selectivity filter as they have been postulated for AA [25]. The intactness of carboxyl head of the molecule was another structural need since major alterations as in anandamide and 2-arachidonoylglycerol let to a loss of inhibitory efficacy (see figure 1 for structures and scheme of blocking efficacy of the fatty acids). However, detailed structural analysis on yet not available crystal structures of the open and closed K_Ca_3.1 channel and mapping of AA and EETs interaction/binding will be needed to provide more definite insight into this lipid modulation of K_Ca_3.1 channels. In contrast to eicosanoids and ω3, the pentacyclic triterpenes studied here did not modulate mK_Ca_3.1 channel, which might be explained by their more “rigid” and larger structures that may not fit into the internal cavity of the channel.

From the physiological and pharmacological perspective, mircomolar EETs, stable EET-analogues, and 20-HETE have been used to study mechanisms of vasodilation or vasoconstriction. Since K_Ca_3.1 has been demonstrated a major component in the EDH-mediated type of endothelium-dependent vasodilation [59] and considering that this channel modulates also functions in several other tissues [1], [2], interactions of the different EET and of 20-HETE with K_Ca_3.1 channels as described in the present study needs to be taken into account.

An additional interesting observation was that 5,6-EET was capable to antagonize AA-inhibition of K_Ca_3.1-activity in isolated patches (Figure 4). Moreover, at the whole-cell level, 5,6-EET potentiated Ca^2+^-pre-activated K_Ca_3.1-currents. While 5,6-EET did not have a direct effect on channel-gating per se as concluded from the inside-out experiments (Figure 3), it was tempting to speculate that 5,6-EET antagonized the -at least partial - channel inhibition caused by endogenous Ca^2+^-dependent PLA_2_-mediated AA-release. This view was fostered by the insensitivity of the hK_Ca_3.1^V275A^ to 5,6-EET-potentiation (Figure 6). Such a mechanism may represent a novel mechanism of endogenous K_Ca_3.1-modulation beyond Ca^2+^-regulation of the channel. Moreover, the 5,6-EET-mediated de-blockade of K_Ca_3.1 could be a thus far unrecognized mechanism underlying EDH-mediated vasodilation, in which both EETs and K_Ca_3.1 have been implicated to play major roles.

It is worth to mention that K_Ca_3.1 channels contribute to a variety of pathologies such as acute and chronic inflammation [60], [61], vasculo-occlusive disease (neointima formation) [12], atherosclerosis [62], angiogenesis [22], polycystic kidney disease [63], ulcerative colitis [21], [64], tumor growth and metastasis (e.g. glioblastoma [17]), transplant vasculopathy [65], [66], and organ fibrosis [67]. EETs, ω3, and pentacyclic triterpenes have also been reported to mechanistically contribute to/influence such disease states [31], [32], [33], [34], [35], [36], [37], [38]. In this respect, some of the reported anti-inflammatory, vaso-protective, and anti-cancerogenic actions of EETs and ω3 as well as anti-hypotensive actions of 20-HETE, but possibly not that of pentacyclic triterpenes, could be explained by inhibition of pro-proliferative K_Ca_3.1 functions. This also raised the possibility to use stable 14,15-EET or 20-HETE mimetics [68] to target K_Ca_3.1 in disease states, to which this channel adds patho-mechanistically.

In conclusion, the present electrophysiological and structure-activity-relationship study demonstrated modulation of cloned and endogenous K_Ca_3.1 channels by selective EETs, 20-HETE, and ω3 and revealed major structural determinants of the molecules for channel interaction.
